# Association of different pathologic subtypes of growth hormone producing pituitary adenoma and remission in acromegaly patients: a retrospective cohort study

**DOI:** 10.1186/s12902-021-00850-2

**Published:** 2021-09-16

**Authors:** Maryam Dehghani, Zahra Davoodi, Farahnaz Bidari, Amin Momeni Moghaddam, Davood Khalili, Hooman Bahrami-Motlagh, Elena Jamali, Shahram Alamdari, Farhad Hosseinpanah, Mehdi Hedayati, Majid Valizadeh

**Affiliations:** 1 Prevention of Metabolic Disorders Research Center, Research Institute for Endocrine Sciences, Shahid Beheshti University of Medical Sciences, Tehran, Iran; 2 Department of Endocrinology, Skull Base Research Center, Loghman-Hakim Hospital, Shahid Beheshti University of Medical Sciences, Tehran, Iran; 3 Department of Pathology, Loghman-Hakim Hospital, Shahid Beheshti University of Medical Sciences, Tehran, Iran; 4 Department of Radiology, Taleghani Hospital, Shahid beheshti University of Medical Sciences, Tehran, Iran; 5 Department of Radiology, Loghman-Hakim Hospital, School of Medicine, Shahid Beheshti University of Medical Sciences, Tehran, Iran; 6 Obesity Research Center, Research Institute for Endocrine Sciences, Shahid Beheshti University of Medical Sciences, Tehran, Iran; 7 Cellular & Molecular Endocrine Research Center, Research Institute for Endocrine Sciences, Shahid Beheshti University of Medical Sciences, Tehran, Iran

**Keywords:** Acromegaly; pathological subtypes, Biochemical remission, Medical response

## Abstract

**Background:**

Regarding the inconclusive results of previous investigations, this study aimed to determine the association between pathology, as a possible predictor, with remission outcomes, to know the role of pathology in the personalized decision making in acromegaly patients.

**Methods:**

A retrospective cohort study was performed on the consecutive surgeries for growth hormone (GH) producing pituitary adenomas from February 2015 to January 2021. Seventy-one patients were assessed for granulation patterns and prolactin co-expression as dual staining adenomas. The role of pathology and some other predictors on surgical remission was evaluated using logistic regression models.

**Results:**

Among 71 included patients, 34 (47.9%) patients had densely granulated (DG), 14 (19.7%) had sparsely granulated (SG), 23 (32.4%) had dual staining pituitary adenomas. The remission rate was about 62.5% in the patients with SG and DG adenomas named single staining and 52.2% in dual staining groups. Postoperative remission was 1.53-folds higher in the single staining adenomas than dual staining-one (non-significant). The remission rate was doubled in DG group compared to two other groups (non-significant). By adjusting different predictors, cavernous sinus invasion and one-day postoperative GH levels decreased remission rate by 91% (95% CI: 0.01–0.67; *p* = 0.015) and 64% (95% CI: 0.19–0.69; *p* < 0.001), respectively. Responses to the medications were not significantly different among three groups.

**Conclusion:**

Various pathological subtypes of pituitary adenomas do not appear to have a predictive role in estimating remission outcomes. Cavernous sinus invasion followed by one-day postoperative GH is the strongest parameter to predict biochemical remission.

## Introduction

Acromegaly is a clinical syndrome caused by excess growth hormone (GH) production almost always from a GH-secreting pituitary adenoma. Despite low prevalence, the disorder has received more attention in the clinical setting because of its high morbidity and mortality [1–5]. Therefore, early diagnosis and appropriate treatment approaches have an especial role to reduce complications, and mortality can be decreased to the level of normal population [6]. The predictive role of age, tumor size, as well as pre-operative GH and IGF-1 levels on remission outcomes are reported in previous studies, and some studies suggested that the clinicopathological information of acromegaly patients may be able to predict the success rate of treatment which provide individualized modalities in therapeutic process [7]. Hence, one of the possible factors which are sometimes considered as remission predictor is pathologic subtypes. Based on the GH granulation pattern in immunohistochemistry (IHC), two pathologic subtypes of GH-producing adenomas are densely granulated (DG) and sparsely granulated (SG) adenomas. About 30–50% of acromegaly patients have DG-GH adenomas with a diffuse positivity for GH [8–10]. They are diagnosed at older ages (> 50 years) which slowly grow [11–15]. It was reported that DG-GH adenomas have an indolent course and are more responsive to surgery and somatostatin analogs than SG-one [12, 16–18]. It is reported that 15–35% of acromegaly patients have SG-GH adenomas with a focal and weak positivity for GH, and they are more common in younger ages (< 50 years) [7]. Some studies indicated that these tumors are more invasive which have a higher ki-67 level [19], larger size, lower GH and IGF-1 levels which are more frequently clinically subtle [12, 13, 15, 20–23].

These two pathological subtypes considered single staining adenomas, approximately a quarter of somatotropic adenomas also prolactin (PRL)-positive, so-named dual staining (or mixed GH-PRL) adenomas [24]. These adenomas are composed of mixed somatotroph-lactotroph tumors, mammosomatotroph tumors, and more primitive and aggressive tumors such as acidophil stem cells [25, 26]. The new important topic at recent clinicopathological discussion is that dual staining tumors compared with pure somatotropic-ones may have a different treatment and risk of invasion to surrounding tissues [7, 10].

As far as we know, limited previous studies assessed the pathological diversities among different pituitary somatotroph adenomas, and dual staining tumor etiologies are reported to some extent, but their clinical impact is not clear [27]. Thus, the role of pathology, as a predictor factor, is inconclusive and needs more investigation. We investigated this knowledge gap by comprehensive comparison among different pathologic subtypes of pituitary adenomas in terms of clinical manifestations, laboratory, imaging findings, and especially treatment outcomes.

## Materials and methods

### Study population & design

Shahid Beheshti University of Medical Sciences ethically approved the present study under the code of IR.SBMU.ENDOCRINE.REC.1400.016. The informed consent form was gathered from all patients. All methods were carried out based on STROBE guidelines. A retrospective review of 89 consecutive surgeries for GH-producing pituitary adenomas performed from February 2015 to January 2021 was undertaken. All these Endoscopic Trans Sphenoidal Surgeries (ETSS) were performed at a single tertiary referral center (Loghman Hospital). Acromegaly was diagnosed based on clinical features and increase in serum IGF1 levels and non-suppressible GH after Oral Glucose Tolerance Test (OGTT). Demographic, clinical, biochemical, and radiological data were extracted from hospital registry system. Loghman hospital has had a registry system for pituitary tumors since 2015. Acromegaly patients were included who had at least three-month follow-up information after surgery, and pathological specimens were available for more evaluations. Pregnant women, the patients with incomplete information, previous pituitary surgery or radiotherapy, and history of treatment with somatostatin analogs, tumor morphology with predominant cystic appearance and necrosis, as well as pituitary hemorrhage were excluded from this study. Considering these criteria, 71 patients were included in this investigation. Given the retrospective nature of this study and anonymized patient data, the patient consent was not needed for this study.

### Biochemical evaluation

Hormonal profiles of sera, including GH, IGF-1, and other hormones of pituitary axis including prolactin, thyroid stimulating hormone (TSH), thyroxine (T4), cortisol, adrenocorticotropic hormone (ACTH), testosterone, follicle-stimulating, and Luteinizing hormone (FSH and LH) were assessed before and after surgery in fasting state by a patented Electro Chemi Luminescence (ECL) technology.

Serum GH levels were recorded one day after surgery, and other pituitary axis was assessed 1 and 3 months or more later. To evaluate the patient’s biochemical remission, serum IGF-1 and random GH were measured, and if needed, GH after oral glucose tolerance test (OGTT) was assessed 3–6 months postoperatively. Basal IGF-1 and random GH were recorded 3 and 6 months after medical treatment to evaluate medical responsiveness and then whenever clinically required. For GH after OGTT, after an overnight fasting serum, GH and blood glucose were measured in the morning. Then, the patients received 75 g glucose load and serum GH, as well as blood glucose, was measured after 30, 60, and 120 min.

All results obtained by Cobas e 611 analyzers is a fully automated analyzer (Roche Diagnostics, Indianapolis, Indiana, USA), except IGF-1 which uses an immunodiagnostic systems analyzer (IDS-iSYS; Boldon Business Park, Boldon, Tyne & Wear, NE35 9PD, UK) with reportable range 10 to 1200 ng/ml and CV total 7.2% and CV within run 1.4% for mean 304 ng/ml.

### Neuroimaging examination

All included patients underwent preoperative Magnetic Resonance Imaging (MRI) at the time of diagnosis during follow-up. Tumor size before surgery (micro or macro-adenoma), tumor extension (to the cavernous sinus, infra sellar, or suprasellar), and knosp classification were recorded. The cavernous sinus invasion was considered positive in the patients with Knosp grades 3 and 4. Evidence of tumor remnant 3 months after surgery and/or MRI imaging postoperatively was defined as subtotal resection. For this investigation, MRI images of some available patients were reviewed again to determine T2 intensity by an experienced radiologist. Classifying T2 MRI intensity of a pituitary adenoma was assessed by qualitative analysis (visual assessment method). The adenoma is classified as hypointense (if the signal appears less intense than the gray matter), isointense (as intense as gray matter), and hyperintense (more intense than gray matter) [28].

### Histopathologic evaluation

For this investigation, the tissues were extracted from pituitary archives based on their registry code, and their specimens were sectioned (5 μm) and prepared on poly L-lysine coated slides. Slides were immunohistochemically stained based on Master Polymer Plus Detection system (Peroxidase Ind. DAB Chromogen, MAD-0000237-QK) for Growth Hormone, Prolactin, and Ki-67 using monoclonal antibodies (mAbs) against Rabbit anti-human GH (Clone EP 267 Master Diagnostica), Rabbit anti-human Prolactin (Clone EP 193 Master Diagnostica) and Ki-67 Antibody & Probe (BRB040-Z-1 and BRB040–3), respectively. The evaluation of each slide using a light microscope (Nikon, Tokyo, Japan) which was independently done by two well-experienced pathologists who did not know the patients’ characteristics and clinical outcome. Discrepancies were solved via discussion, and κ test was calculated to determine agreement percentage. Finally, clinical and paraclinical features before surgery, as well as surgical outcomes and response to medical therapy, were compared among different pathological subtypes. Once we compare single and dual staining group staining as a binary comparison, and again we compare DG, SG, and dual staining groups as a triple comparison.

### Surgical outcomes

According to recent guidelines, biochemical remission criteria were defined as IGF-1 normalization adjusted to age and gender, and GH levels below 1 ng/ml, 3–6 months after surgery [29, 30]. The response to first generation-SRL was assessed 3–6 months after starting medication by IGF-I levels and classified into three different groups. (1) full responders, defined as normal age-matched IGF-I levels; (2) partial responders, defined as IGF-I reduction ≥50% from baseline but without normalization and (3) poor responders, defined as IGF-I reduction < 50% from baseline [31].

Recurrence was defined as IGF-1 levels increase to greater than normal after initial normalization [32]. Other complications, including cerebrospinal fluid leak, meningitis, focal neurological deficit, and hypopituitarism, were evaluated after surgery. Hypopituitarism after surgery, including central adrenal insufficiency, central hypogonadism, central hypothyroidism, and diabetes insipidus were defined according to recent clinical practice guidelines [33].

### Statistical analyses

All normally distributed continuous variables were expressed as mean ± standard deviation (SD). Otherwise, skewed-distributed continuous variables were shown as median and inter-quartile range (IQR) 25–75, and qualitative variables as number and percentage. To compare baseline characteristics of single and dual staining adenomas for quantitative variables with normal distribution, we used a two-tailed, independent sample *t*-test. Nonparametric variables were analyzed using Mann–Whitney test. Qualitative variables were analyzed with Chi-square and Fisher’s exact tests. Characteristics across three pathological groups, DG, SG, dual staining, were compared using analysis of variance, Kruskal-Wallis H, and chi-square test for normal, skewed, and categorical variables, respectively. Random effect models assist to control heterogeneity caused by the presence of different surgeons.

Odds ratios (ORs) of remission with 95% confidence intervals (CIs), 6 months after surgery were estimated for different pathological groups and some other predictors using univariate and multivariate logistic regression models. All analyses were performed using STATA software, version 14, and *p*-values < 0.05 were considered significant.

## Results

### Baseline characteristics

Descriptive analysis of preoperative baseline characteristics of each group is shown in Table 1. Seventy-one included patients were divided based on the pathologic subtypes of pituitary adenoma in two and three groups. The single staining group consisted of 48 (% 67.6), and dual staining consisted of 23 (32.4%) patients. Total mean age was 41.4 ± 10.7 years, with a 62% female distribution. About 86% of tumors were macro adenomas and the mean size of adenomas was 18.8 ± 10 mm. The rate of supra sellar extension, infra sellar, and cavernous sinus invasion by the tumor was 69, 31, and 22.9%, respectively. T2 MRI intensity was reported iso or hyper-intense, and hypo-intense. After calling by researcher, only 34 patients brought us their MRI for T2 intensity assessment. In total, 67.6 and 32.4% of patients were iso or hyper-intense and hypo-intense, respectively. Twenty-three percent of specimens were Ki-67 positive more than 3%, and the mean of Ki-67 antibody was 1.93 ± 1.45. About 27% of patients were p53 positive. The kappa test was calculated to determine the percentage of agreement between two pathologists, it was 0.87 (*p* = 0.001). There was no significant difference between single and dual staining groups in preoperative clinical presentations, biochemical profiles, MRI findings, and proliferative indices.

**Table 1:** Baseline characteristics of various subtypes of GH- producing pituitary adenoma patients

Variables	Single Staining *n* = 48 (%67.6)			Dual Staining *n* = 23(%32.4)	Total *n* = 71(100%)
	Densely granulated *n* = 34 (%47.9)	Sparsely granulated *n* = 14 (%19.7)	Total *n* = 48 (%67.6)		
Demographic and Clinical data					
Mean age, yrs. (SD)	40.9 ± 11.0	40.3 ± 10.9	40.7 ± 10.9	42.8 ± 10.3	41.4 ± 10.7
Females, n (%)	22(64.7)	9(64.3)	31(64.6)	13(56.5)	44(62.0)
Body mass index, mean (SD)	27.1 ± 3.5	29.9 ± 5.9	27.9 ± 4.5	28.1 ± 3.9	28.0 ± 4.3
Clinical manifestations, n (%)					
Acromegalic Features	30(88.2)	13(92.9)	43(89.5)	21(91.3)	64(90.1)
Headache	18(52.9)	8(57.1)	26(54.2)	16(69.6	42(59.2)
Oligoamenorrhea	11(50.0)	5(55.6)	16(51.6)	7(53.8)	23(52.3)
Weight gain	15(44.1)	10(71.4)	25(52.1)	11(47.8)	36(50.7)
Decreased libido	16(47.1)	7(50)	23(47.9)	10(43.5)	33(46.5)
Visual Complaint	13(38.2)	6(42.9)	19(39.6)	13(56.5)	32(45.1)
Hyper hydrosis	13(38.2)	3(21.4)	16(33.3)	7(30.4)	23(32.4)
Hypertension	9(26.5)	2(14.3)	11(22.9)	6(26.1)	17(23.9)
Diabetes mellitus	4(11.8)	3(21.4)	7(14.6)	5(21.7)	12(16.9)
Weakness	4(11.8)	3(21.4)	7(14.6)	2(8.7)	9(12.7)
Galactorrhea	5(14.7)	1(7.1)	6(12.5)	2(8.7)	8(11.3)
Hypopituitarism, n (%)					
Central hypogonadism	19(55.9)	10(71.4)	29(60.4)	14(60.9)	43(60.6)
Central hypothyroidism	1(2.9)	2(14.3)	3(6.2)	0(0)	3(4.2)
Central adrenal insufficiency	1(2.9)	0(0)	1(1.4)	0(0)	1(1.4)
Biochemical profile (Before surgery)					
Median prolactin level, ng/ml (IQR)	21(11.9–34.2)	25(17.2–43)	24(13.1–34)	32(14–56)	25(13.4–40.0)
Prolactin elevated, n (%)	18(52.9)	9(64.3)	27(56.2)	14(60.9)	41(57.7)
Median IGF-1 level, ng/ml (IQR)	615(524.7–784.7)	692.5(470.2–950.5)	631.5(520.2–839.5)	651(439–860)	633(488–850)
Mean GH level, ng/ml (SD) ^†^	17.6 ± 17.1	11.5 ± 5.9	15.8 ± 14.9	14.6 ± 14.5	15.4 ± 14.7
MRI Tumor characteristics					
Size					
Mean diameter, mm (SD)	17.9 ± 9.0	24.4 ± 10.9	19.8 ± 9.9	16.97 ± 10.1	18.8 ± 10.0
Macroadenoma, n (%)	29(85.3)	13(92.9)	42(87.5)	19(82.6)	61(85.9)
Size-group > 17 mm, n (%)	17(50.0) *	11(78.6) *	28(58.3)	8(34.8) *	36(50.7)
Extrasellar extension					
Suprasellar extension, n (%)	24(70.6)	12(85.7)	36(75)	13(56.5)	49(69.0)
Cavernous sinus invasion, n (%)	6(18.2)	6(42.9)	12(25.5)	4(17.4)	16(22.9)
Infrasellar invasion, n (%)	7(20.6)	6(42.9)	13(27.1)	9(39.1)	22(31.0)
T2- MRI Intensity, n (%)					
Iso or hyper-intense‡	10(58.8)	7(87.5)	17(68.0)	6(66.7)	23(67.6)
Hypo-intense	7(41.2)	1(12.5)	8(32.0)	3(33.3)	11(32.4)
Pathological Tumor characteristics					
Mean of Ki-67 (SD)	1.80 (1.59)	2.33 (1.40)	1.96 (1.54)	1.84 (1.21)	1.93 (1.45)
Ki-67 > 3% (SD), n (%)	6(18.8)	5(41.7)	11(25.0)	3(17.6)	14(23.0)
P_53_ Positive^††^, n (%)	6(21.4)	6(60)	12(31.6)	3(17.6)	15(27.3)

*SD* standard deviation. *IQR* interquartile range

‡ The Iso or hyper-intense subgroups were combined into a single group because of the low sample size in hyper intense subgroup

^†^_Mean GH levels adjusted for age, sex, and BMI_

^††^_P53 was evaluated for 55 patients_

*Significant difference among the Single Staining Densely granulated, Single Staining Sparsely granulated, and Dual Staining groups (*p* < 0.05)

** Significant difference between the Single Staining, and Dual Staining groups (*p* < 0.05)

### Surgical outcomes

One-day postoperative GH and complications are summarized in Table 2. During the median 12(6,24) months follow-up, 33 (46.5%) patients had central hypogonadism, 5(7%) had permanent DI, 22 (30%) central hypothyroidism, and 6 (8.5%) patients had central adrenal insufficiency. No mortality was recorded in this series of patients. There was no significant difference between single versus dual staining groups in the surgical complications. The surgeon was included the model as a random effect but had no significant effect (*p*-value = 0.450).

**Table 2:** Comparison of surgical outcomes among various subtypes of GH- producing pituitary adenoma

Variables	Single Staining *n* = 48 (%67.6)			Dual Staining *n* = 23(%32.4)	Total *n* = 71(100%)
	Densely granulated *n* = 34 (%47.9)	Sparsely granulated *n* = 14 (%19.7)	Total *n* = 48 (%67.6)		
Random GH levels, ng/ml (IQR) ^†^	1.3(0.7–2.5)	1.45(0.95–6.62)	1.3(0.7–3.5)	1.69(0.85–3.22)	1.5(0.7,-3.5)
Median follow-up, month (IQR)	12(6,24)	12(6,15)	12(6,24)	12(6,24)	12(6,24)
Hypopituitarism, n (%)					
Central hypogonadism	15(44.1)	9(64.3)	24(50)	9(39.1)	33(46.5)
Diabetes insipidus	15(44.1)	7(50.0)	22(45.8)	9(39.1)	31(43.7)
Central hypothyroidism	9(26)	7(50)	16(33)	6(26)	22(30)
Central adrenal insufficiency	3(8.8)	2(14.3)	5(10.4)	1(4.3)	6(8.5)
Cerebrospinal fluid leak, n (%)	3(8.8)	3(21.4)	6(12.5)	0(0)	6(8.5)
Meningitis, n (%)	0(0)	1(7.1)	1(2.1)	0(0)	1(1.4)
Focal neurological deficit, n (%)	0(0)	0(0)	0(0)	0(0)	0(0)

*IQR* interquartile range

† One day post-operative-GH levels adjusted for age and sex, BMI

*Significant difference among the Single Staining Densely granulated, Single Staining Sparsely granulated, and Dual Staining groups (*p* < 0.05)

** Significant difference between the Single Staining, and Dual Staining groups (*p* < 0.05)

### Disease remission

**Fig. 1:**
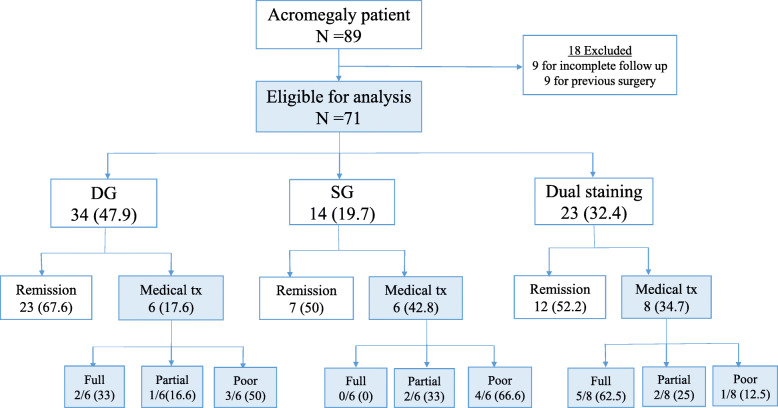
The overall rate of postoperative remission, response to medical treatment, according to the pathological subtypes. Remission criteria were defined as the IGF-1 normalization, adjusted to age, sex, and GH levels below 1 ng/ml, 3–6 months after surgery [29, 30]. Medical tx refers to the response to first generation-SRL assessed 3–6 months after starting medication by IGF-I levels and classified in three different groups. Full responders, defined as normal age-matched IGF-I levels; partial responders, defined as IGF-I reduction ≥50% from baseline but without normalization; poor responders, defined as IGF-I reduction of < 50% from baseline [31]

**Fig. 2:**
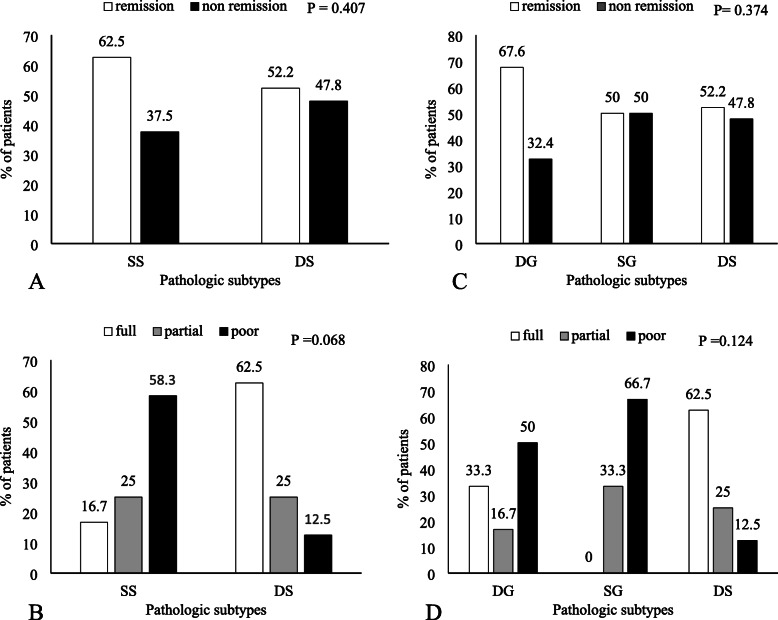
Bar graphs. The remission rate and medical responsiveness in various pathological subtypes. Panel-**A** showing no pathological subtypes, single staining (SS), or dual staining (DS) was predictive of surgical remission. Panel-**B** showing no pathological subtypes, single staining (SS), or dual staining (DS) was predictive of medical responsiveness. Panel-**C** showing no pathological subtypes, densely granulated (DG), sparsely granulated (SG), or dual staining (DS) was predictive of surgical remission. Panel-**D** showing no pathological subtypes, densely granulated (DG), sparsely granulated (SG), or dual staining (DS) was predictive of medical responsiveness

Total resection was performed in 62% of patients, and postoperative biochemical remission was achieved in 59.2% (42/71). During follow-up period, 27 patients received medical treatment, one of three patients achieved disease control with cabergoline, and considering the response to Sandostatin LAR, 7 patients were full, 5 patients were partial, and 8 patients were poor responders. Therefore, 8 patients responded to medical treatment completely. 5(11.9%) patients experienced recurrence after surgery, thus in the end, 63.3% (45/71) patients had a controlled disease. According to pathological subtypes, the overall rate of postoperative remission and response to medical treatment are shown in Fig. 1.Table 3 shows the postoperative remission rate in various pathological subtypes. The remission rates were 62.5% (30/48) in single and 52.2% (12/23) in dual staining group. There was no statistically significant difference between single and dual staining groups (*p* = 0.407), (Fig. 2A).

**Table 3:** Comparison of biochemical remission and medical responsiveness among various pathological subtypes of GH-producing pituitary adenoma

Variables	Single Staining *n* = 48 (%67.6)			Dual Staining *n* = 23(%32.4)	Total *n* = 71(100%)
	Densely granulated *n* = 34 (%47.9)	Sparsely granulated *n* = 14 (%19.7)	Total *n* = 48 (%67.6)		
Remission after surgery					
Total resection, n (%)	25(73.5)	6(42.9)	31(64.6)	13(56.5)	44(62.0)
Biochemical remission, n (%)	23(67.6)	7(50.0)	30(62.5)	12(52.2)	42(59.2)
Responses to medications					
^†^Medication for remission, n (%)	9(26.5)	8(57.1)	17(35.4)	10(43.5)	27(38.0)
Mean Sandostatine LAR dosages, mg (SD)	23.3 ± 5*	30 ± 5.8*	26.2 ± 6.2	22.2 ± 4.4*	24.6 ± 5.9
Response to Sandostatine LAR, n (%)					
Full responder	2(33.3)	0(0)	2(16.7)	5(62.5)	7(35.0)
Partial responder	1(16.7)	2(33.3)	3(25.0)	2(25.0)	5(25.0)
Poor responder	3(50.0)	4(66.7)	7(58.3)	1(12.5)	8(40.0)
Recurrence^††^, n (%)	0(0) *	3(42.8) *	3(10)	2(16.6) *	5(11.9)
Resurgery, n (%)	1(2.9)	2(14.3)	3(6.2)	0(0)	3(4.2)
Radiation, n (%)	2(5.9)	3(21.4)	5(10.4)	0(0)	5(7.0)

*SD* standard deviation. *IQR* interquartile range

†Medication for remission: Cabergoline & Sandostatine LAR

†† Recurrence was evaluated for 42 patients who achieved remission after surgery

*Significant difference among the Single Staining Densely granulated, Single Staining Sparsely granulated, and Dual Staining groups (*p* < 0.05)

** Significant difference between the Single Staining, and Dual Staining groups (*p* < 0.05)

Remission was not achieved in 40.8% (29/71) of patients. Twenty patients were treated with Sandostatin LAR, and there was no significant difference between single and dual staining in medical responsiveness (*p* = 0.068), (Table 3 and Fig. 2B). Furthermore, the mean dosage of Sandostatin LAR was similar in single and dual staining groups, 26.2 ± 6.2 and 22.2 ± 4.4, respectively (*p* = 0.100).

In the logistic regression of surgical cure, following predictors were assessed in univariate analysis: pathological subtypes, age, sex, pre and post-operative GH and IGF1, tumor size, cavernous sinus invasion, and T2 MRI intensity, Ki-67 and p53 (Table 4). The single staining adenomas have 1.53-folds as much improvement in disease remission after surgery compared with the dual staining-one, but there was no statistical difference among them (*p* = 0.409). After adjusting for all variables in multivariate analysis, only cavernous sinus invasion reduced disease remission by 91% (95% CI: 0.01–0.67) and 1 day postoperative GH for each nanogram per milliliter, reduced disease remission by 64% (95% CI: 0.19–0.69).

**Table 4:** Logistic regression for determining possible predictive factors on surgical remission in the GH-producing pituitary adenoma

Variable		OR (%95 CI)		
	*p*-value	Univariate	Multivariate1	Multivariate2
Pathology subtypes				
Densely granulated	0.242	1.92 (0.64–5.69)	3.21 (0.53–19.30)	
Sparsely granulated	0.898	0.92 (0.24–3.46)	0.94 (0.09–10.20)	
Dual Staining		1	1	
Pathology subtypes				
Single Staining	0.409	1.53 (0.56–4.17)		2.41(0.46–12.48)
Dual Staining		1		1
Pathology subtypes				
Densely (reference: S)	0.255	2.09(0.59–7.45)		
Some other predictors				
Age	0.298	1.02 (0.98–1.07)		
Sex (reference: Male)	0.051	2.68 (1.00–7.20)	6.91 (1.08–44.28)	5.13 (0.96–27.40)
Pre-operative GH	**0.028**	**0.95 (0.91–0.99)**	0.94 (0.87–1.01)	0.95 (0.88–1.02)
Pre-operative IGF1	0.537	1.00 (0.99–1.00)		
One-day postoperative GH	**< 0.001**	**0.36 (0.21–0.62)**	**0.33 (0.17–0.67)**	**0.36 (0.19–0.69)**
Tumor diameter (mm)	0.318	0.98 (0.93–1.02)		
Tumor diameter (youden index:1.7)	0.269	0.583(0.22–1.52)		
Cavernous sinus invasion	**0.015**	**0.23 (0.07–0.75)**	**0.09 (0.01–0.70)**	**0.09 (0.01–0.67)**
T2 MRI intensity (reference: iso or hyper)	0.264	0.42 (0.09–1.91)		
Ki-67 (reference: ≤3)	0.437	0.62 (0.19–2.06)		
P53	0.221	0.47 (0.14–1.57)		
Extention of surgery (reference: total resection)	**< 0.001**	**0.016 (0.004–0.073)**		
Skill of Surgeon	**0.450**	1.99(0.33–11.88)		

Significant variables at the 0.20 alpha level in the univariate analysis were included in the multivariate models. Since the pathology of GH-producing pituitary adenomas is a clinically important determinant of remission,in this study. This factor was included in multivariate1 as a three-subtype predictor and in multivariate2 as a two-subtype predictor

Moreover, we compared all above variables among DG, SG, and dual staining groups as a triple comparison, and the most important results were as follows: First, tumor sizes over 17 mm were more frequent in SG group about 78.6% compared with two other groups, 50, and 34.8% respectively. Second, although there was no statistically significant difference in remission rate among DG, SG, and dual staining groups (*p* = 0.374) (Fig. 2C), DG adenomas have two-fold more remission rates compared to other groups (*p* > 0.05) (Table 4). And third, considering the response to medical treatment, the mean dosage of Sandostatin LAR was higher in SG than the other groups, 30 ± 5.8 in SG but lower in DG and dual staining group 23.3 ± 5 and 22.2 ± 4.4, respectively (*P* = 0.013). But there was no statistically significant difference in medical responsiveness among DG, SG, and dual staining groups (*P* = 0.124) (Fig. 2D).

## Discussion

Acromegaly is a slowly progressive disease, and identifying the predictors in the clinical course of disease determines the next step in decision making. We investigated the correlation of histological parameters as a possible predictor, on biochemical remission, for knowing the role of pathology in personalized treatment plan in acromegaly. In our series of acromegaly patients, different pathological subtypes of pituitary adenomas appear do not have a predictive role to estimate biochemical remission, both in binary and triple comparisons. After comparing the various predictors, our results showed that the cavernous sinus invasion and preoperative and one-day postoperative GH have a significant association with remission in acromegaly patients. After adjusting all variables, the cavernous sinus invasion as key factor decreased the remission rate by 91%, followed by one-day postoperative GH, about 64%. After adjusting all variables, the cavernous sinus invasion and one-day postoperative GH levels showed a significant predictive role in the patient’s remission.

In evaluating various predictive factors on remission, most studies emphasize the importance of tumor invasion and GH level, our study is in line with them [34, 35]. Moreover, a systematic review by Agrawal et al. [32] showed that among predictors of surgical outcomes, some of them consistently reduce remission rate in almost all studies such as cavernous sinus invasion, tumor size, and higher GH levels, some of them occasionally reduce remission rates. In our study, after adjusting for covariates, the best single preoperative predictor was cavernous sinus invasion, followed by GH level in the first few days postoperatively. On the other hand, although tumor sizes were higher in SG group, remission rate was not statistically significant among three groups.

Similar to the findings of Rick et al. [27] study, in our results, tumor size showed no significant association with disease remission, also in Kreutzer et al. [36] study, the significance of tumor size on disease remission was borderline.

Studies about prolactin co-expression of GH-producing pituitary adenomas as a remission predictor have had heterogeneous results; some studies showed prolactin co-expression is a negative remission predictor [27, 36, 37], some showed it has not predictive role [38–40], and some mentioned prolactin co-expression is positive remission predictor [41]. In this regard, our findings are similar to Sun et al. [38], their study was done on 59 acromegaly patients that pathologically classified into three groups SG, DG, and dual staining pituitary adenomas, and they found that surgical remission was similarly about 50% in each group. Minniti et al. [40] also found similar results indicating no prognostic value for dual staining adenoma. However, the results of Rick et al. study were different [27], they reported that single-staining tumors have8.6 times more remission rate than dual-staining tumors, they emphasized that prolactin staining is a major predictor of surgical remission in acromegaly. We think their results need to interpret cautiously because the odds ratio has a wide confidence interval (1.09–109.7). On the other hand, they did not evaluate the different subtypes of single staining group, including the percentage of SG adenoma, also their single staining group had fewer acromegalic features, lower IGF-1, prolactin levels, and smaller tumor size compared with dual staining one. These findings were different from our study which indicated an earlier diagnosis of acromegaly in a single staining group may influence their conclusion. Marinis et al. [37] found that high serum prolactin levels and dual staining pathology negatively affect remission outcomes. However, half of the patients in the remission group (controlled patients) had microadenomas, and on the opposite side, sellar floor perforation and destruction were more frequent (16/25) among non-remission (poorly controlled) patients, which may influence their conclusion.

As we mentioned, the results of different studies contradict the remission rate in dual staining compared with single staining adenomas. There are several hypotheses to justify this result heterogeneity; first, it is possible that single staining identified falsely as dual staining adenomas, and vice versa. Dual staining adenomas are identified when prolactin expression is present in a higher percentage of cells (more than 5%) than pure single staining adenomas [42], but there is uncertainty in practice due to the semi-quantitative nature of IHC method. On the other hand, like any other neuroendocrine tumor, somatotroph adenomas, may intermittently produce prolactin, and they may indicate a wide spectrum of differentiation and variably express prolactin that causes pathological subtypes misclassification, too [43]. Second, studies which used electron microscopy and molecular analysis showed that dual staining adenomas compose of mammosomatotroph tumors and Mixed Somatotroph-Lactotroph, which may have different behavior. Mammosomatotroph tumors look like DG adenomas in IHC method, but they also express estrogen receptor (ER) and prolactin [44]. They are very similar to DG adenomas clinically but they may have more significant hyperprolactinemia [10]. A mixed Somatotroph-Lactotroph tumor consists of two separate cell populations, somatotrophs, and lactotrophs. Each cell population can be DG or SG, so various combinations may occur. The characteristics of these tumors are dependent on how to combine the relative proportions of tumor cells [44]. Hence, depending on which tumors and cell populations cause prolactin immune-reactivity in dual staining adenomas, clinical features, and remission response may be different in various studies. Third, remission criteria in acromegaly didn’t have the same references in various studies, and this caused a patient in different studies to be placed in different remission categories.

Our study indicated no statistically significant difference in remission rate among DG, SG, and dual staining groups.

Medical responsiveness results are less heterogeneous than surgical remission among various pathological subtypes, considering a biological rationale about different SSTR2 expressions and medical responsiveness in acromegaly. SRL responsiveness was assessed among various pathological subtypes, the response rate in SG was lower than in two other groups; however, a non-significant association may be due to the small sample size. In Brzana et al. [13] study, like ours, SG group was less responsive to the medical treatment. Therefore, the tumor behavior and clinical characteristics of dual-stained adenomas are more similar to sparsely granulated adenomas in contrast with our study, this can be due to the different composition of cell composition in dual staining groups. Recurrence, re-surgery, and radiation were higher in SG compared to two other groups; however, only recurrence rate was statistically significant. This finding is also in contrast with Brzana study.

Regarding the strengths of present study, it was a single-center study which was the referral hospital of pituitary diseases. Two well-experienced pathologists reported the results of pathology specimens, separately with an acceptable agreement. Besides, patient’s responses to surgery and medical therapy were assessed according to the newest criterion [31]. Ultimately, this is the first time that in a single study, the pathology of pituitary adenoma was analyzed in two separate strata, single and dual staining in binary comparison, on the one hand, and on the other, triple comparison among DG, SG, and dual staining. However, the study has some limitations. The retrospective nature of study which is often of limited value and patients, did not follow up actively, so some data were missed due to un-accessibility. Second, the sample size was small which can affect our result in various pathological subtypes. Third, the type of cell population, the mammosomatotroph or mixed-somatotroph-lactotroph, in dual staining adenomas had not been determined separately by the electronic microscope because these two subtypes may have different behavior and response to treatment.

In conclusion, our findings do not support a major predictive role of different pathological subtypes of pituitary adenomas to estimate treatment response. However, among all predictors we analyzed, the cavernous sinus invasion and one-day GH level post-operative seem strongly associated with treatment outcomes. Inconclusive results of previous studies about pathologic subtypes, maybe originate from cell population misclassification, different remission criteria. Further studies with more accurate tools such as electron microscopes as well as newer molecular markers are needed to certain the prognostic role of the pathology on the treatment response in acromegaly patients.

## Data Availability

The datasets used and/or analysed during the current study available from the corresponding author on reasonable request.
